# RDCTrans U-Net: A Hybrid Variable Architecture for Liver CT Image Segmentation

**DOI:** 10.3390/s22072452

**Published:** 2022-03-23

**Authors:** Lingyun Li, Hongbing Ma

**Affiliations:** 1 College of Information Science and Engineering, Xinjiang University, Urumqi 830046, China; lingyun_li1823@163.com; 2 Beijing National Research Center for Information Science and Technology, Department of Electronic Engineering, Tsinghua University, Beijing 100084, China

**Keywords:** liver tumor segmentation, U-Net, ResNeXt50, dilated convolution, transformer

## Abstract

Segmenting medical images is a necessary prerequisite for disease diagnosis and treatment planning. Among various medical image segmentation tasks, U-Net-based variants have been widely used in liver tumor segmentation tasks. In view of the highly variable shape and size of tumors, in order to improve the accuracy of segmentation, this paper proposes a U-Net-based hybrid variable structure—RDCTrans U-Net for liver tumor segmentation in computed tomography (CT) examinations. We design a backbone network dominated by ResNeXt50 and supplemented by dilated convolution to increase the network depth, expand the perceptual field, and improve the efficiency of feature extraction without increasing the parameters. At the same time, Transformer is introduced in down-sampling to increase the network’s overall perception and global understanding of the image and to improve the accuracy of liver tumor segmentation. The method proposed in this paper tests the segmentation performance of liver tumors on the LiTS (Liver Tumor Segmentation) dataset. It obtained 89.22% mIoU and 98.91% Acc, for liver and tumor segmentation. The proposed model also achieved 93.38% Dice and 89.87% Dice, respectively. Compared with the original U-Net and the U-Net model that introduces dense connection, attention mechanism, and Transformer, respectively, the method proposed in this paper achieves SOTA (state of art) results.

## 1. Introduction

Liver cancer is currently one of the most common cancer diseases in the world, causing a large number of deaths every year [1,2]. Liver cancer is a malignant tumor of the liver, which can be classified into two categories: primary and secondary. Primary liver cancer originates from the epithelial or mesenchymal tissue of the liver and is a high-incidence and extremely harmful malignant tumor in China; secondary liver cancer is called sarcoma, which is relatively rare compared with primary liver cancer. As the largest solid organ of the human body, the liver undertakes various important metabolic functions of the human body. Once malignant tumors appear in the liver, they can lead to serious and life-threatening consequences. Therefore, early detection and treatment are the keys to improving the survival rate of liver cancer patients. CT-based imaging methods are commonly used to evaluate liver tumors, and CT examinations can clearly show the size and shape, number, and boundaries of lesions. Segmentation of liver lesions is a preparatory step for diagnosis and plays an indispensable role in the treatment of the disease. Liver segmentation is divided into manual segmentation and semi-automatic segmentation. However, manual segmentation largely relies on the judgment of radiologists, which is time-consuming and error-prone; adding manual intervention in the semi-automatic segmentation process will lead to bias and errors. The task of automatically segmenting liver tumor lesions becomes very challenging given the unique diversity and spread of liver tumor shapes.

In recent years, with the wide application of deep learning technology in medical image segmentation tasks, more and more researchers use deep learning to achieve automatic segmentation of liver tumors [3,4]. However, what really improves the performance of liver tumor segmentation is the U-Net proposed by Ronneberger et al. [5]. U-Net is developed based on the FCN [6] proposed by Long et al. in 2015, which makes up for more low-level semantic information with its unique U-shaped and skip connection structure, and it only needs to be trained with a small amount of data to obtain more accurate results. With the development of medical technology, the U-net architecture is widely used for various medical imaging analysis. However, as a 2D network, the U-Net network has difficulties using the 3D spatial information of liver slices and cannot automatically segment 3D liver images such as CT and MRI [7]. To solve this problem, Ahmed et al. proposed 3D U-Net [8] in 2016, which replaced all 2D operations with corresponding 3D operations on the basis of U-net architecture to produce 3D segmented images, effectively using the spatial information between adjacent liver slices and achieving better segmentation results. In 2016, Liu et al. [9] designed a network suitable for 3D medical image segmentation by introducing the residual connection idea of Res-Net [10] on the basis of U-Net, where the encoder is used to extract liver features and the decoder is used to generate full-resolution output. This method improves segmentation accuracy but requires longer training time due to the large number of parameters for 3D convolution. To this end, Li et al. [11] proposed H-Dense U-Net in 2018 to alleviate this problem. The idea is to mix the features of 2D Dense U-Net and 3D Dense U-Net to accelerate the convergence of 3D Dense U-Net, while embedding dense connection blocks in U-Net, which can further improve the segmentation accuracy of liver and tumor. In 2017, Han et al. [12] proposed to segment liver and tumor by stacking multiple 2D information into 3D information. The method is based on U-Net’s skip connections and Res-Net’s residual connections, and then, it provides 3D contextual information by inputting multiple consecutive single slices. However, the acquisition of 3D contextual information by adding densely connected and residually connected models is limited. Therefore, in 2020 Cai et al. [13] combined local features with their corresponding global dependencies by adding attention gates (AG) [14]. In the liver tumor segmentation task, it is guaranteed that it automatically ignores other irrelevant regions while focusing only on the liver tumor location and explicitly modeling the dependencies between channels helps to capture rich contextual dependencies. It is worth mentioning that in 2017, Vaswani et al. [15] proposed a network based entirely on the attention mechanism and successfully applied it to the NLP field, and brought profound inspiration to scholars in the CV field. Because compared to Attention, the parallel ability of Transformer’s own self-attention enables it to have better adaptability in the face of big data. In addition, the Transformer model is flexible and can be applied to any type of data if it is abstracted as a series of embeddings. Therefore, in 2021, Dosovitskiy et al. [16] used Transformer directly in the image block sequence to complete the task of image classification, successfully achieved the most advanced performance on multiple image recognition benchmarks, and successfully applied Transformer to the field of computer vision. In the same year, Chen et al. [17] proposed that Transformer cannot be used purely in image segmentation tasks. After being inspired by the ViT architecture, the Transformer encoder was introduced into U-Net for down-sampling. Using Transformer to solve the long-distance dependence of a convolutional network increases the network’s overall perception and global understanding of the image. At the same time, combined with U-net, it can enhance finer details by recovering local spatial information. Compared with other Transformer models such as VIT, Trans U-Net not only shows better segmentation performance in liver, stomach, and other multi-organ segmentation but is also the first visual Transformer model for medical image segmentation.

Inspired by the above, a model named RDCTrans U-Net (ResNeXt50-Dilated Convolution-Transformer U-net) was proposed. The main contributions of this paper are as follows:

1. ResNeXt can solve the gradient problem caused by reducing the increase of residual connections, so this paper uses ResNeXt50 as the down-sampling backbone to increase the depth of the network without increasing parameters.
2. Dilated convolution is an effective kernel for adjusting the receptive field of feature points without reducing the resolution of feature maps. Therefore, this paper replaces all 3 × 3 convolutions in the last layer (Bottleneck × 3) of ResNeXt50 with dilated convolutions and improves the segmentation performance by increasing the receptive field.
3. Since the Transformer can capture global information, this paper introduces the Transformer part in the encoder part to enhance the global context encoding ability of the overall structure and the ability to distinguish semantics.

Experiments conducted on a partial LiTS dataset show that the network in this paper has superior performance on the liver segmentation task compared to U-Net and some of its variants. We demonstrate the coordination and efficiency of ResNeXt50, dilated convolution, and Transformer in down-sampling tasks through ablation experiments.

## 2. Related Work

### 2.1. U-Net

In the context of medical images, the image is expensive and complex to acquire, and this also adds to the complexity of accurately annotating images [18]. However, CNNs have shown great potential in medical image segmentation in recent years [8,19], most of which is attributed to U-Net [5]. The structure of U-Net is very similar to Seg-Net [20], consisting of an encoder and a decoder. The difference lies in the skip connections between the encoder and decoder in each layer. The architecture and data expansion of U-Net allows the learning model to have a very good generalization performance from only a few annotated samples [21]. It has become a practical standard for medical image segmentation even when the amount of labeled training data are limited [10].

U-Net is derived from the idea of improving FCN [6], but it has many improvements compared to FCN. First of all, U-Net is completely symmetric, up-sampling uses adjacent interpolation, and the decoder is processed by convolution and deepening. Second, skip connections combine global and local features to form thicker features. Finally, U-Net uses valid convolution throughout to ensure segmentation results without missing contextual features. Because U-Net performs multi-scale fusion, combines low-resolution information and high-resolution information, and provides the basis for object category identification and accurate segmentation and positioning, it is very suitable for medical image segmentation.

### 2.2. Res-Net

The traditional convolutional network has the problem of information loss during information transmission, and it also causes the gradient to disappear or the gradient to explode, making the deep network unable to train. Res-Net [10] solves this problem to a certain extent. Its main idea is to add a direct channel to the network and protect the integrity of the information by directly detouring the input information to the output. Compared with VGG-Net [22], the biggest difference between Res-Net is that there are many bypasses to directly connect the input to the following layers. This structure is also called shortcut.

**Figure 1:**
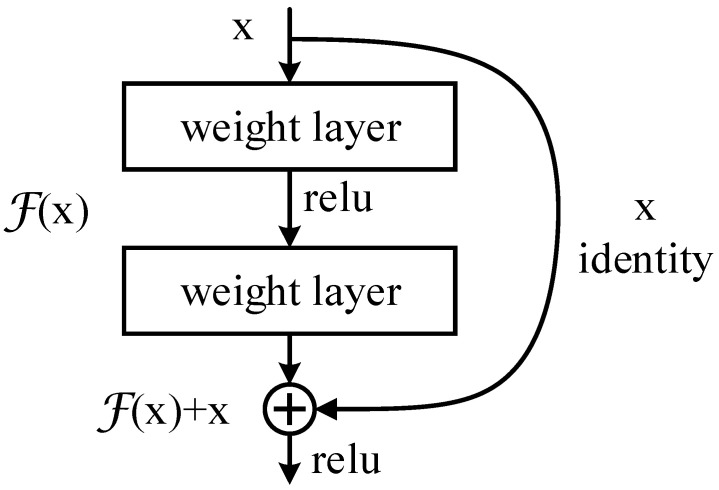
Residual structure.

The residual structure is shown in Figure 1. By adding the identity mapping, the original function H(x) that needs to be learned is converted into F(x)+x*,* that is, H(x)=F(x)+x, and the entire network only needs to learn the part of the difference between input and output. This idea stems from residual vector encoding in image processing. Through an information reorganization, the input and output of this module are superimposed at the element level. Not only does it not add extra parameters and computation to the network, but it can greatly increase the training speed of the model and improve the training effect.

### 2.3. ResNeXt

Although the proposal of the residual structure solves the problem of gradient disappearance caused by the deepening of the network layer, the modules of the same topology are stacked in Res-Net. This makes each component of the entire network more cumbersome, and the properties of the branches contained in each component are more variable. Inspired by the structure of the Inception series of networks [23,24], the ResNeXt [25] structure is designed, combining it with the residual structure in Res-Net, and simplifying the branch design method in Inception, making it modular. It can improve accuracy without increasing parameter complexity while reducing the number of hyperparameters. Especially when depth and width start to bring diminishing returns to existing models, increasing cardinality in ResNeXt is a more effective way to improve accuracy than increasing depth and width. Here cardinality is the size of the transformation set, a specific, measurable dimension of central importance.

### 2.4. Dilated Convolution

Common image segmentation algorithms usually use pooling and convolutional layers to obtain multi-scale contextual information. The pooling operation generally reduces the feature map size (resolution) first and then uses up-sampling to restore the image size. Although the receptive field of the neural network can be effectively increased, this operation of scaling down and then scaling up will lead to problems such as reduced resolution of feature maps and loss of spatial information. Therefore, there is a need for an operation that can increase the receptive field while keeping the size of the feature map unchanged, so as to replace the down-sampling and up-sampling operations. Under this requirement, dilated convolution was proposed by Yu et al. [26]. Dilated convolution can detect and segment large objects by expanding the receptive field without losing resolution, and increasing the resolution can precisely locate objects without introducing additional parameters or computational cost [27]. Different from the normal convolution, the dilated convolution introduces a hyper-parameter called “Hole Size”, which can get different perceptual field sizes by setting the number of holes, thereby capturing multi-scale context information and generating large-scale feature maps with rich spatial information, which can be effectively applied in the field of semantic segmentation.

### 2.5. Transformer

With recent advances in NLP research, some segmentation methods have explored alternatives based on channel or spatial [28,29] attention and pointwise [30] attention to better capture contextual information. However, these methods still rely mainly on convolutions and thus are more biased towards local interactions. The usual use of specialized layers to compensate for this bias shows the limitations of convolutional architectures for segmentation [28,31]. To overcome these limitations, Vaswani et al. [15] formulate the semantic segmentation problem as a sequence-to-sequence problem and exploit contextual information at each stage of the model using a Transformer architecture, which is entirely based on Attention [14]. Transformer uses Positional Encoding to understand the order of language, self-attention mechanism (Self Attention Mechanism), and fully connected layer for calculation, which is a typical encoder-decoder structure. But the biggest difference compared with traditional CNN is its parallel training, which can greatly improve computational efficiency.

The great success of Transformers in NLP has also influenced the CV field [32,33]. In various medical image segmentation tasks, the success brought by the use of the U-Net architecture has become a reality. However, due to the inherent local nature of convolutional operations, U-Net typically exhibits limitations in explicitly modeling remote dependencies. This problem is addressed by the proposal of Trans U-Net [17]. Transformer, as a powerful encoder for medical image segmentation tasks, is combined with U-Net to enhance finer details by recovering local spatial information. In different medical applications for multi-organ segmentation, Trans U-Net achieves performance superior to various competing methods.

## 3. Methods

**Figure 2:**
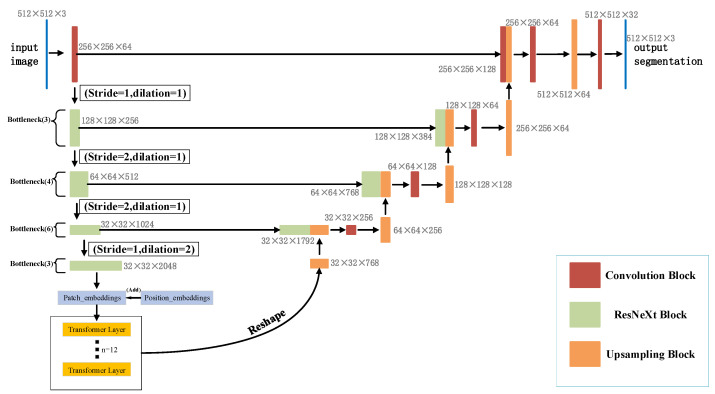
Network structure diagram of RDCTrans U-Net.

**Figure 3:**
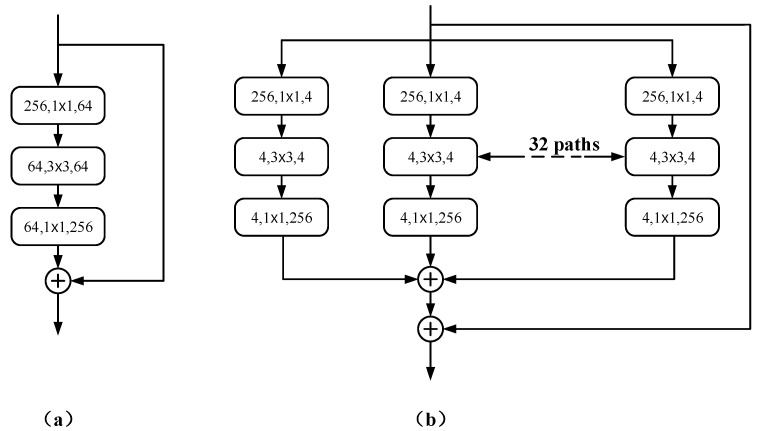
(**a**) An early version of Res-Net; (**b**) an aggregation block of ResNeXt50.

**Figure 4:**
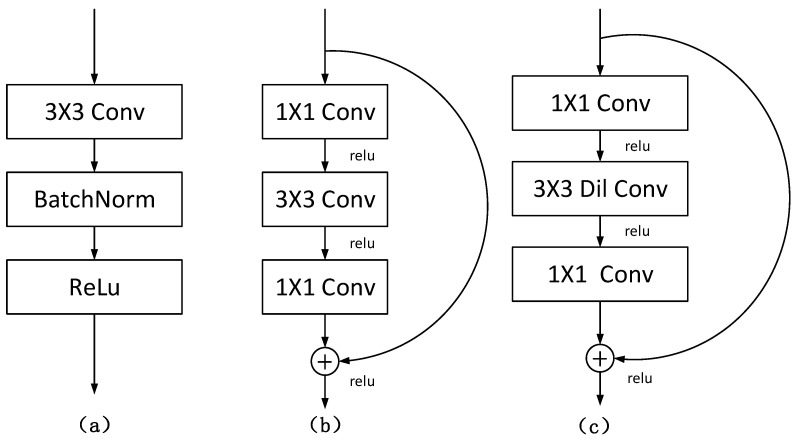
(**a**) The structure of convolution block; (**b**) the original structure of Bottleneck; (**c**) the structure of Bottleneck replaced with dilated convolution.

In this paper, a new segmentation architecture-RDCTrans U-Net (ResNeXt50-Dilated Convolution-Transformer U-Net) is proposed. The network structure is shown in Figure 2**.** In the encoder part, we use ResNeXt50 as shown in Figure 3b to extract feature maps and adopt skip connections in the U-Net structure to combine the multi-path feature maps of the mid-layer and deep layers, while using dilated convolution to refine the deep feature map of the fourth block of ResNeXt and fuse the global context information. The decoder part consists of four modules; each module contains one up-sampling block and two convolutional blocks; each convolutional block consists of convolutional layer, Batch Normalization, and ReLU. The structure is shown in Figure 4a.

### 3.1. U-Net

U-Net [5] is divided into a down-sampling stage and an up-sampling stage, and the two stages use the same number of layers of convolution operations. The skip connection structure can connect the down-sampling layer with the up-sampling layer. After the channel features are extracted from the down-sampling layer, they can be directly transferred to the up-sampling layer, which greatly improves the segmentation accuracy. There is no fully connected layer in the network structure, and the shallow and deep layers are used to solve the problems of pixel localization and pixel classification, respectively, so as to achieve image semantic level segmentation.

In the experiment, in order to enhance the U-Net structure, the U-Net encoder part is improved into a hybrid encoder composed of three modules: ResNeXt50, dilated convolution, and Transformers, which are described in detail below.

### 3.2. ResNeXt50 

ResNeXt [25] was created based on the idea of stacked networks such as Res-Net. It first aggregates a set of transformations with the same topology, then uses residual connections to augment blocks of multiple convolutional layers and generates gradient shortcuts that greatly reduce the risk of vanishing gradients, thus allowing training of deeper network structures.

Inspired by ViT [16], the ResNeXt50 encoder is used as the backbone to enhance the encoder structure of the baseline U-Net, where 50 represents the number of layers. The basic structure of the aggregation block in ResNeXt50 is shown in Figure 3b.

Following the highly modular design rule of Res-Net [10], it is only necessary to design template modules to determine all modules in the network. RDCTrans U-Net consists of one convolutional block and four residual blocks with the same topology. To adapt to the segmentation task, the global average pooling layers and fully connected layers in ResNeXt50 are removed. The Rectified Linear Unit (ReLU) [34] of Batch Normal (Batch Norm) [35] is added to the first convolution block. Batch Norm can make the network converge faster so that both the training set and the test set can remain independent and identically distributed and alleviate the overload problem of data initialization. The introduction of ReLU can solve the gradient vanishing problem of activation function backpropagation [36] in deep networks and alleviate the problem of network overfitting. The latter four residual blocks we replace are composed of bottleneck layers (Bottleneck) [37] with 3, 4, 6, and 3 stacked blocks, respectively, as shown in Figure 2. The use of bottleneck layers enables the reduction of parameters, making it possible to train and extract features from the data more efficiently and intuitively after dimensionality reduction. The cardinality of the aggregated blocks in ResNeXt50 is set to 32, as shown in Figure 3b.

### 3.3. Dilated Convolution

Dilated convolution [26] is a new type of convolution that allows aggregating multi-scale context. Dilation rates using kernels k and l of size M means sampling the input image with a stride of *l*, as shown in Equation (1):

(1) $$y\left[i,j\right]=\sum_{n=1}^{M}\sum_{m=1}^{M}x\left[i+l*m,j+l*n\right]k\left[m,n\right]$$

All 3 × 3 convolutions in the last bottleneck layer of the ResNeXt50 structure are replaced by displacement-0 dilated convolutions with constant kernel size and dilation rate l=2, as shown in Figure 4c. This operation can refine the fourth deep feature map, and the receptive field can be increased without changing the size of the feature map, so that ResNeXt50 can capture a wider range of contexts, and then better integrate global information.

### 3.4. Transformers

The feature maps learned from ResNeXt50 are divided into a series of patches. To better utilize the Transformer to learn location information, a learnable location embedding is performed on each patch to obtain the location matrix of N patches.

**Figure 5:**
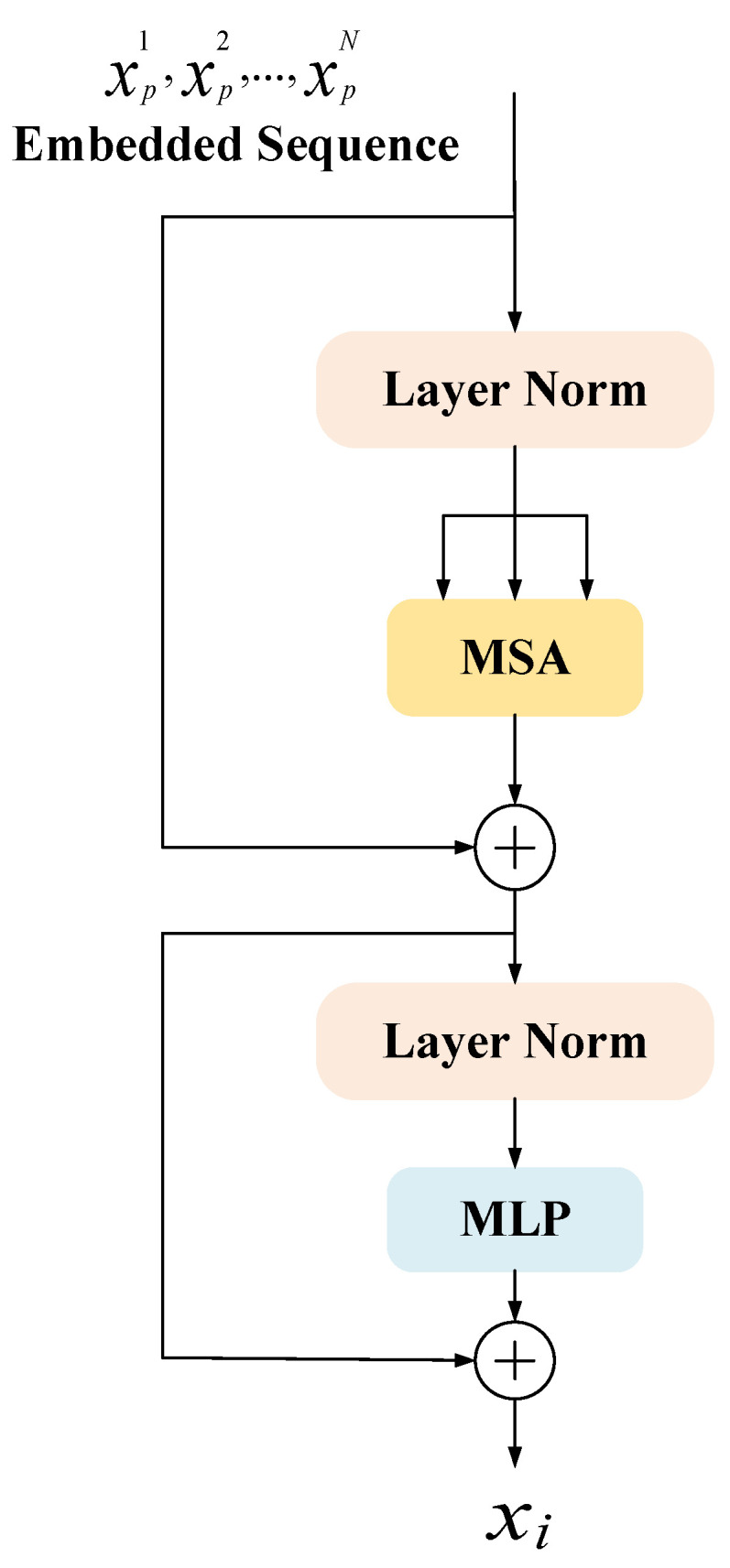
Encoder schematic of Transformer.

Transformers here is a collection of 12 concatenated Transformer encoders. The structure of a single Transformer encoder is shown in Figure 5, which is mainly composed of three parts: multi-head self-attention (MSA), multi-layer perceptron (MLP), and layer normalization (Layer Norm).

Multiheaded Self-Attention (MSA) can obtain more levels of semantic information and can reduce the total amount of calculation by reducing the dimension. It is essentially multiple independent Attention [14] calculations, and the role of integration is to prevent over-fitting. The definition of Multiheaded Self-Attention is shown in Equations (2) and (3).

(2) $$MultiHead\left(Q,K,V\right)=Concat\left(head_{1},\ldots ,head_{h}\right)W^{o}$$

(3) $$head_{i}=Attention\left(QW_{i}^{Q},KW_{i}^{K},VW_{i}^{V}\right)$$

where Q, K, and V are single inputs from the same data; $W^{Q}$, $W^{K}$, and $W^{V}$ are the weight matrices obtained by linear(∗) transformation with three different parameters, and Concat denotes integration. i represents the number of multi, i=8.

MLP (Multi-Layer Perceptron) contains three parts: input layer, hidden layer, and output layer, and the purpose is to realize the mapping from input to output. Each layer of it is fully connected to the next layer, which is called the fully connected layer, and the definition of the fully connected layer is shown in Equation (4).

(4) $$FFN\left(x\right)=\max\left(0,xW_{1}+b_{1}\right)W_{2}+b_{2}$$

The fully connected layer here is a two-layer neural network that first maps the input *Z* to a higher-dimensional space, linearly transforms it, filters it through the nonlinear function ReLU, and then linearly transforms it to the original dimension.

Layer normalization (Layer Norm) [38] is to normalize all neurons in an intermediate layer, which can alleviate the problem of gradient disappearance and explosion in the early stage of training and improve stability. As shown in Equation (5).

(5) $${\tilde{z}}^{\left(l\right)}=\frac{z^{\left(l\right)}-\mu^{\left(l\right)}}{\sqrt{\sigma^{\left(l\right)}+\epsilon }}⊙\gamma +\beta ⇐LN_{\gamma ,\beta }\left(z^{\left(l\right)}\right)$$

where $z^{\left(l\right)}$ is the net input of the l-th layer of neurons, $\mu^{\left(l\right)}$ and $\sigma^{\left(l\right)}$ are its mean and variance, respectively, and γ,β represent the scaling and translation parameter vectors; the dimension is the same as $z^{\left(l\right)}$. 

After the Transformer structure is introduced into the down-sampling part, it is convenient to use the parallelized training of Transformer to better capture full-text information. The architecture is entirely based on attention, which can suppress irrelevant background and highlight useful features, which helps to localize tumors quickly and accurately in the task of liver tumor segmentation.

## 4. Experiments

We used the LiTS public dataset for training to obtain a segmentation model for liver tumors. Compared with four advanced segmentation methods (U-Net [5], Attention U-Net [14], Dense U-Net [11], and Trans U-Net [17]), the method in this paper has the best segmentation results. We verify the effectiveness of our proposed model with comparative experiments on the LiTS dataset, including two ablation analysis experiments, as detailed in Section 4.4.

### 4.1. Datasets and Metrics

To validate the performance of our model in liver tumor segmentation, we use the MICCAI 2017 Liver Tumor Segmentation Challenge (LiTS) dataset [39] as the experimental dataset.

The segmentation of liver and its lesions in medical images is helpful for accurate diagnosis and therapeutic evaluation of liver cancer. The LiTS dataset includes 200 CT scans provided by clinical sites around the world, and images from each CT sequence provide liver and lesion areas through masks. CT imaging consists of three processes: First, an X-ray scan is obtained and converted into digital information. Then, the voxels are generated and separated by a computer, and the X-ray coefficients of each voxel are obtained and arranged into a digital matrix. Finally, the digital matrix corresponds to different grayscales according to the values of its entries, and a CT grayscale image is obtained. In the LiTS dataset, each CT scan image contains a large number of axial slices, typically ranging from a few hundred to thousands, with an axial slice resolution of 512 × 512 pixels; the labels are divided into 3 categories: background (label 0), liver (label 1), liver tumor (label 2).

To train our proposed model, we randomly sampled 1371 2D slice images containing liver tumors from this dataset, where 1096 images are used as training set, 131 images are used as validation set, and 144 samples are used as test set. All images are normalized using Equation (6) to improve the overall training process speed.

(6) $$value_{normalized}=\frac{value_{original}-mean}{std}$$

where $value_{original}$ and $value_{normalized}$ show the original image pixel value and the normalized image pixel value, respectively. Mean represents the mean value of the image pixels, and std represents the standard deviation of the image pixels. In addition, in order to satisfy the training of neural network and prevent over-fitting during model training, we also performed data flipping and data scaling operations on data images for data enhancement.

In the work covered in this paper, we use Mean Intersection over Union (*mIoU*) and Accuracy (*Acc*) to evaluate the comprehensive segmentation performance of our proposed model. Additionally, we use the Dice Similarity Coefficient (DSC), Precision (*Pr*), and Recall (*Re*) to evaluate the segmentation performance of the proposed model for liver and tumor, respectively.

*mIoU* is the arithmetic mean of pixel-level intersection/union (*IoU*) [40] and *n* test image samples, defined as Equation (7). *Acc* is the accuracy rate [41], which refers to the proportion of correctly predicted pixels of the category (background class and target class) to the total image pixels, as shown in Equation (8). The Dice Similarity Coefficient (DSC) [42] is a measure of the ensemble similarity, as shown in Equation (9). Precision (*Pr*) is the ratio of the number of correctly predicted positive samples to the total number of predicted positive samples, as shown in Equation (10). Recall (*Re*) is the ratio of the number of correctly predicted positive samples to the total number of actual positive samples, defined as Equation (11). The larger the value of these five indicators, the better the model segmentation performance.

(7) $$mIoU_{i}=\frac{1}{n}IoU_{i}=\frac{1}{n}\sum_{i=1}^{n}\frac{TP}{TP+FP+FN}$$

(8) $$Acc=\frac{TP+TN}{TP+TN+FP+FN}$$

(9) $$Dice=\frac{2TP}{FP+2TP+FN}$$

(10) $$Pr=\frac{TP}{TP+FP}$$

(11) $$Re=\frac{TP}{TP+FN}$$

where TP, TN, FP, and FN are pixel-level metrics representing the true, true negative, false positive, and false negative values in the confusion matrix, respectively.

The loss function used in the medical segmentation task in this paper is cross-entropy (*CE*) [43] defined as Equation (12).

**Figure 6:**
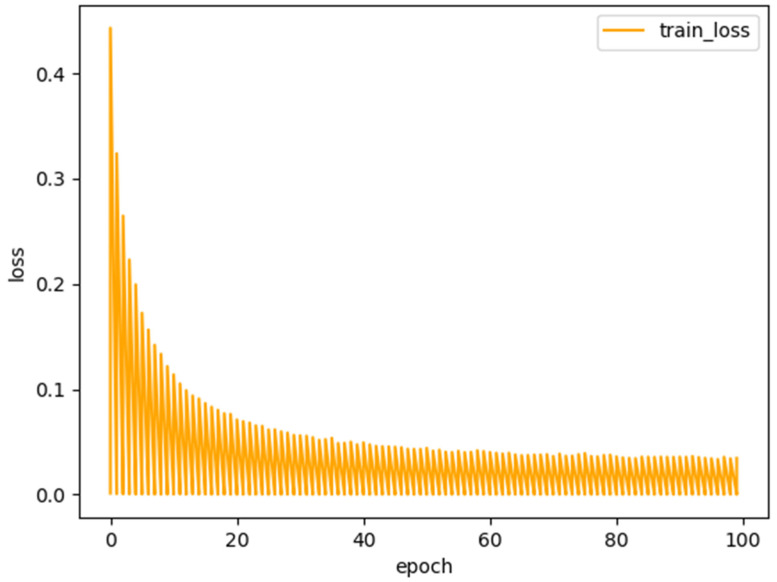
Loss function of the proposed model RDCTrans U-Net.

(12) CE(A,B)=−(Alog(B))+(1−A)log(1−B)

where B and A represent the predicted and underlying ground-truth splits, respectively. The loss function curve of the proposed model RDCTrans U-Net is shown in Figure 6. It can be seen that after 100 training iterations, the loss value is reduced to 0.1, indicating that the trained deep learning network model has converged.

### 4.2. Experimental Details

In this experiment, the proposed RDCTrans U-Net is implemented in Python using the Pytorch deep learning framework. In addition, we use stochastic gradient descent (SGD) [44] with a batch size of 2, momentum of 0.9, and weight decay of 5 × 10^−4^ instead of Adam optimization [45], which according to a recent study [46] shows that SGD usually leads to better performance, although Adam optimization converges faster. We trained the model using a fixed size training image (512 × 512) and trained 100 batches on an NVIDIA GeForce RTX 1080 Ti GPU. For a fair comparison, the parameters of all experiments were set to the same case. During the training process, the model that performs best on the validation set is selected as the final model. We use cross-entropy loss (*CE*) as the loss function to optimize the model.

### 4.3. Comparative Experiments

To verify the validity of the proposed model, we selected the original U-Net method and three recent popular U-Net variant models (Attention U-Net, Dense U-Net, and Trans U-Net) for comparison. In the stage of evaluating the model performance, firstly, in the LiTS dataset, we adopted the evaluation metrics of common medical image segmentation tasks—*Acc* (Accuracy) and *mIoU* (mean Intersection over Union), to verify the overall segmentation performance of our proposed model RDCTrans U-Net and the comparison model. The experimental results are shown in Table 1. Second, we use Dice similarity coefficient (DSC), precision rate (*Pr*), and recall rate (*Re*) to verify the segmentation performance of these models in the LiTS dataset for liver and tumor, respectively. The experimental results are shown in Table 2.

As can be seen from Table 1, RDCTrans U-Net achieves the best performance on the LiTS dataset, with an *mIoU* value of 89.22% and an *Acc* of 98.91%. This is 14.93% higher than the *mIoU* value of the baseline method Original U-Net, and also much higher than the *mIoU* value of some classic U-Net variant methods such as Attention U-Net, Dense U-Net, and Trans U-Net. They are 6.13%, 10.04%, and 5.9% higher, respectively. Furthermore, compared with these classical methods, the *Acc* metric of our proposed model RDCTrans U-Net is 2.26% higher than the traditional U-Net, reaching 98.91%. This verifies the effectiveness of our proposed U-Net improved model.

As can be seen from Table 2, for liver segmentation, the Dice score, Precision, and Recall of the proposed model RDCTrans U-Net reach 93.38%, 88.65%, and 98.89%, respectively, which achieves the best liver segmentation performance compared to these contrasting algorithms. Meanwhile, for tumor segmentation, the Dice score and Precision of our model are higher than those of all contrasting models. It is worth mentioning that the Recall of the proposed model is higher than that of other models except that the Recall is lower than that of Attention U-Net by about 1%. This proves that the proposed model has an ideal segmentation effect in both liver and tumor.

To compare the complexity of each model, all models were trained on NVIDIA GeForce RTX 1080 Ti GPUs. Among them, the training time of Trans U-Net, Attention U-Net, and the proposed model RDCTrans U-Net is about 20 h, the training time of Dense U-Net is about 23 h, and the training time of Original U-Net is about 16 h. Obviously, our model complexity is higher than the Original U-Net, but compared to these U-Net variant models, the model complexity does not increase. Through the analysis, it is concluded that ResNeXt is a structure that improves the accuracy without increasing the complexity of the parameters. Using it as the down-sampling backbone network will not increase the complexity of the model, while the complexity of Transformer is quadratically related to the input sequence, which leads to higher model complexity of the proposed model and Trans U-Net.

### 4.4. Ablation Experiments

To further determine the effectiveness of our proposed method, we performed two experimental analyses of ablation. During the ablation analysis, we only use the two metrics *Acc* and *mIoU* to evaluate the impact of each module on the model segmentation performance. Table 3 shows the comprehensive segmentation performance of the proposed model RDCTrans U-Net and two ablation models on the LiTS dataset.

First, we remove the dilated convolution and Transformer modules and only use ResNeXt50 as the encoder for down-sampling, and we name the network model ResNeXt U-Net. Second, we only remove the Transformers module, combine ResNeXt50 and dilated convolution as the encoder part of the model, and name the model Dilated ResNeXt U-Net.

From Table 1 and Table 3, we can see that ResNeXt U-Net achieves *mIoU* of 80.92% and *Acc* of 96.79% on LiTS dataset. Although it is not as good as the comprehensive segmentation effect of Attention U-Net, it is better than the comprehensive segmentation effect of the original U-Net, Dense U-Net and ResNeXt U-Net network. This proves the effectiveness of replacing the traditional CNN encoder with the Resnext50 encoder, which improves the segmentation accuracy of the model to a certain extent. To further expand the receptive field of the network, we add dilated convolution to the last bottleneck layer. The *mIoU* of Dilated ResNeXt U-Net on the LiTS dataset is 83.15%, and the *Acc* is 97.32%, which proves that adding dilated convolution can effectively improve the performance of the network.

In addition, Table 3 shows that RDCTrans U-Net achieves an *mIoU* of 89.08% and an *Acc* of 98.91% on the LiTS dataset. Comparing RDCTrans U-Net with ResNeXt U-Net and Dilated ResNeXt U-Net, our proposed model has the best overall segmentation performance. This further proves the effectiveness of our proposed hybrid encoder, and at the same time verifies that the multi-head self-attention in Transformers can effectively obtain global context information, making up for the shortcomings of convolution operations in this regard. In the encoding part, the combination of ResNeXt, dilated convolution, and Transformers can make the network segmentation of medical images more accurate.

### 4.5. Visual Analysis

**Figure 7:**
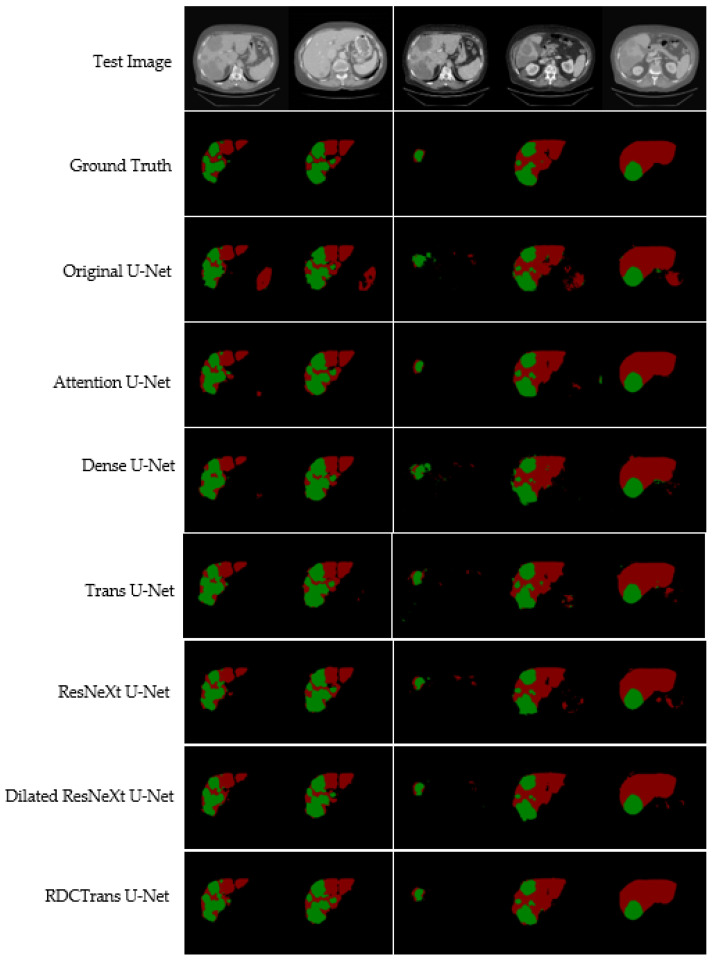
Illustrates the liver and tumor segmentation results of different methods on the test dataset. The red area represents the liver, and the green area represents the tumor.

We propose a deep learning model RDCTrans U-Net for liver and tumor segmentation, and to verify the effectiveness of our model, we conduct comparative experiments with other state-of-the-art methods. At the same time, the segmentation results of liver tumors were visualized on the LiTS dataset, as shown in Figure 7. We list 5 CT images containing liver and tumor to visually see that the segmentation results of our proposed model are significantly better than other models.

Compared with liver segmentation, liver tumor segmentation is considered to be the most difficult segmentation task due to the variable shape and uncertain size of liver tumors. As can be seen from Figure 7, for the segmentation of liver and tumor, the segmentation images of the proposed model RDCTrans U-Net are closer to the Ground Truth map than those of other models. The original U-Net, Attention U-Net, and Dense U-Net over-segment or under-segment the liver, leading to poor segmentation results, which indicates that the Transformer-based model has stronger global context encoding ability and the ability to distinguish semantics. In addition, the Transformer-based Trans U-Net model also has a good segmentation effect because the Transformer encoder can learn the global contextual feature representation of the image, especially to encode the position information of the image, which definitely helps to improve the overall segmentation effect of the image. However, the segmentation accuracy of our proposed model is higher, which is attributed to the fact that the ResNeXt internal CNN and residual structure are more interested in some finer features on the image, such as tumor edge features. At the same time, we replace the 3×3 convolution inside Bottleneck with dilated convolution to expand the receptive field, which can capture multi-scale context information and further improve the segmentation performance of the model. As can be seen from Figure 7, this paper combines the Transformer module with ResNeXt50 and dilated convolution, which effectively improves the segmentation accuracy of liver and tumor. In addition, Table 2 lists the detailed liver and tumor segmentation results. For liver and tumor segmentation, we achieve 93.38% Dice and 89.87% Dice, respectively, which is an ideal performance for liver and tumor segmentation. These observations demonstrate that RDCTrans U-Net is capable of finer segmentation and preserves detailed shape information.

## 5. Conclusions

In this work, we propose RDCTrans U-Net to meet the need for more accurate liver CT image segmentation tasks. The network structure proposed in this paper is designed based on the U-Net architecture, and only the encoder part is modified. First, down sampling with the ResNeXt50 encoder as the backbone, in which the 3 × 3 convolution in the last bottleneck layer of ResNeXt50 is replaced with a dilated convolution, increases the depth of the network and increases the receptive field. Then through the Transformers encoder integrated structure, the context global information can be effectively obtained, which greatly makes up for the shortcomings of U-Net in convolution. The reconstructed encoder has no pooling layer and fully connected layer, which maximizes the integrity of semantic information, and the results of liver image tumor segmentation are clearer and more accurate. In the training on the LiTS dataset, we not only confirmed the effectiveness of the network in this paper but also proved the coordination and efficiency of ResNeXt50, dilated convolution, and Transformer in down-sampling tasks through ablation experiments. However, RDCTrans U-Net also has some shortcomings. The RDCTrans U-Net proposed in this paper currently only performs segmentation on liver and tumor. In future work, we aim to further improve the structure of the proposed model so that it can be generalized to other medical image segmentation datasets and more flexibly applied to common medical image segmentation tasks to evaluate the segmentation performance of RDCTrans U-Net.

## Figures and Tables

**Table 1:** Comparison of overall segmentation performance of each model on LiTS dataset.

Network Structure	*Acc* (%)	*mIoU* Score (%)
Original U-Net	96.65	74.29
Attention U-Net	98.06	83.09
Dense U-Net	96.93	79.18
Trans U-Net	98.17	83.32
RDCTrans U-Net	98.91	89.22

**Table 2:** The segmentation results of liver and tumor of each model on LiTS data set.

Network Structure	*Dice* (%)		*Pr* (%)		*Re* (%)	
	Liver	Tumor	Liver	Tumor	Liver	Tumor
Original U-Net	83.99	78.01	75.44	68.02	94.73	91.41
Attention U-Net	91.62	89.47	87.13	83.79	98.3	95.35
Dense U-Net	89.24	78.89	84.64	67.98	94.36	93.95
Trans U-Net	89.71	82.62	83.19	73.58	97.34	93.82
RDCTrans U-Net	93.38	89.87	88.65	86.52	98.89	94.31

**Table 3:** Results of the ablation study of the proposed model RDCTrans U-Net.

Network Structure	*Acc* (%)	*mIoU* Score (%)
ResNeXt U-Net	96.79	80.92
Dilated ResNeXt U-Net RDCTrans U-Net	97.32 98.91	83.15 89.22

## Data Availability

We evaluate our algorithm on the public dataset of the MICCAI 2017 Liver Tumor Segmentation Challenge (LiTS). The information link is: https://competitions.codalab.org/competitions/17094 (accessed on 23 February 2022).
